# Power-up: A Reanalysis of 'Power Failure' in Neuroscience Using Mixture Modeling

**DOI:** 10.1523/JNEUROSCI.3592-16.2017

**Published:** 2017-08-23

**Authors:** Camilla L. Nord, Vincent Valton, John Wood, Jonathan P. Roiser

**Affiliations:** ^1^Institute of Cognitive Neuroscience, University College London, London, WC1N 3AZ, United Kingdom, and; ^2^Research Department of Primary Care and Population Health, University College London Medical School, London NW3 2PF, United Kingdom

**Keywords:** neuroscience, power, statistics

## Abstract

Recently, evidence for endemically low statistical power has cast neuroscience findings into doubt. If low statistical power plagues neuroscience, then this reduces confidence in the reported effects. However, if statistical power is not uniformly low, then such blanket mistrust might not be warranted. Here, we provide a different perspective on this issue, analyzing data from an influential study reporting a median power of 21% across 49 meta-analyses (Button et al., 2013). We demonstrate, using Gaussian mixture modeling, that the sample of 730 studies included in that analysis comprises several subcomponents so the use of a single summary statistic is insufficient to characterize the nature of the distribution. We find that statistical power is extremely low for studies included in meta-analyses that reported a null result and that it varies substantially across subfields of neuroscience, with particularly low power in candidate gene association studies. Therefore, whereas power in neuroscience remains a critical issue, the notion that studies are systematically underpowered is not the full story: low power is far from a universal problem.

**SIGNIFICANCE STATEMENT** Recently, researchers across the biomedical and psychological sciences have become concerned with the reliability of results. One marker for reliability is statistical power: the probability of finding a statistically significant result given that the effect exists. Previous evidence suggests that statistical power is low across the field of neuroscience. Our results present a more comprehensive picture of statistical power in neuroscience: on average, studies are indeed underpowered—some very seriously so—but many studies show acceptable or even exemplary statistical power. We show that this heterogeneity in statistical power is common across most subfields in neuroscience. This new, more nuanced picture of statistical power in neuroscience could affect not only scientific understanding, but potentially policy and funding decisions for neuroscience research.

## Introduction

Trust in empirical findings is of vital importance to scientific advancement, but publishing biases and questionable research practices can cause unreliable results (Nosek et al., 2012; Button et al., 2013). In recent years, scientists and funders across the biomedical and psychological sciences have become concerned with what has been termed a crisis of replication and reliability (Barch and Yarkoni, 2013).

One putative marker for the reliability of results is statistical power: the probability that a statistically significant result will be declared given that the null hypothesis is false (i.e., a real effect exists). It can be shown that, in the context of field-wide underpowered studies, a smaller proportion of significant findings will reflect true positives than if power is universally high (Ioannidis, 2005). A recent influential study by Button et al. (2013) calculated statistical power across all meta-analyses published in 2011 that were labeled as “neuroscience” by Thomson Reuters Web of Science. It concluded that neuroscience studies were systematically underpowered, with a median statistical power of 21%, and that the proportion of statistically significant results that reflect true positives is therefore likely to be low. The prevalence of very low power has serious implications for the field. If the majority of studies are indeed underpowered, then statistically significant findings are untrustworthy and scientific inference will often be misinformed. This analysis provoked considerable debate in the field about whether neuroscience does indeed suffer from endemic low statistical power (Bacchetti, 2013; Quinlan, 2013). We sought to add nuance to this debate by reanalyzing the original dataset using a more fine-grained approach and provide a different perspective on statistical power in neuroscience.

We extended the analyses of Button et al. (2013) using data from all 730 individual studies, which provided initial results that were consistent with the original report (which used only the median-sized study in 49 meta-analyses). To quantify the heterogeneity of the dataset we made use of Gaussian mixture modeling (GMM) (Corduneanu and Bishop, 2001), which assumes that the data may be described as being composed of multiple Gaussian components. We then used model comparison to find the most parsimonious model for the data. We also categorized each study based on its methodology to examine whether low power is common to all fields of neuroscience.

We find strong evidence that the distribution of power across studies is multimodal, with the most parsimonious model tested including four components. Moreover, we show that candidate gene association studies and studies from meta-analyses with null results make up the majority of extremely low-powered studies in the analysis of Button et al. (2013). Although median power in neuroscience is low, the distribution of power is heterogeneous and there are clusters of adequately and even well powered studies in the field. Therefore, our in-depth analysis reveals that the crisis of power is not uniform: instead, statistical power is extremely diverse across neuroscience.

## Materials and Methods

### Reanalyzing “power failures”

Our initial analysis took a similar approach to that of Button et al. (2013), but, contrary to their protocol (which reported power only for the median-sized study in each meta-analysis: *N* = 49), we report power for each of the 730 individual studies (see Fig. 3*a*, Table 1). As in the original analysis, we defined power as the probability that a given study would declare a significant result assuming that the population effect size was equal to the weighted mean effect size derived from the corresponding meta-analysis (note that this differs from *post hoc* power, in which the effect size would be assumed to be equal to the reported effect size from each individual study; O'Keefe, 2007).

**Table 1.** Characteristics and classification of included meta-analyses

Study	N	Cohen's *d*	Odds ratio	CI	Significance	Classification
Babbage et al., 2011	13	−1.11		−0.97 to −1.25	*	Psychology
Bai, 2011	18		1.47	1.22–1.77	*	Genetic
Björkhem-Bergman et al., 2011	6	−1.20		1.60–8.00	*	Treatment
Bucossi et al., 2011	21	0.41		0.17–0.65	*	Neurochemistry
Chamberlain et al., 2011	11	−0.51		0.83–1.08	*	Psychology
Chang et al., 2011a	56	−0.19		−0.29 to −0.10	*	Psychology
Chang et al., 2011b	6		0.98	0.86–1.12	—	Genetic
Chen et al., 2011	12		0.60	0.52–0.69	*	Miscellaneous
Chung and Chua, 2011	11		0.67	0.43–1.04	—	Treatment
Domellöf et al., 2011	14		2.12	1.59–2.78	*	Psychology
Etminan et al., 2011	14		0.80	0.70–0.92	*	Treatment
Feng et al., 2011	4		1.20	1.04–1.40	*	Genetic
Green et al., 2011	17	−0.59		−0.93 to −0.26	*	Neurochemistry
Han et al., 2011	14		1.35	1.06–1.72	*	Genetic
Hannestad et al., 2011	13	−0.13		−0.55 to 0.29	—	Treatment
Hua et al., 2011	27		1.13	1.05–1.21	*	Genetic
Lindson and Aveyard, 2011	8		1.05	0.92–1.19	—	Treatment
Liu et al., 2011a	12		1.04	0.88–1.22	—	Genetic
Liu et al., 2011b	6		0.89	0.82–0.96	*	Genetic
MacKillop et al., 2011	57	0.58		0.51–0.64	*	Psychology
Maneeton et al., 2011	5		1.67*^a^*	1.23–2.26	*	Treatment
Ohi et al., 2011	6		1.12	1.00–1.26	*	Genetic
Olabi et al., 2011	14	−0.40		−0.62 to −0.19	*	Brain imaging
Oldershaw et al., 2011	10	−0.51		−0.73 to −0.28	*	Psychology
Oliver et al., 2011	7		0.86	0.79–0.95	*	Treatment
Peerbooms et al., 2011	36		1.26	1.09–1.46	*	Genetic
Pizzagalli, 2011	22	0.92		0.44–1.39	*	Treatment
Rist et al., 2011	5		2.06	1.33–3.19	*	Miscellaneous
Sexton et al., 2011	8	0.43		0.06–0.80	*	Brain imaging
Shum et al., 2011	11	0.89		0.75–1.02	*	Psychology
Sim et al., 2011	2		1.23*^a^*	1.08–1.52	*	Treatment
Song et al., 2011	12	0.15		0.04–0.26	*	Neurochemistry
Sun et al., 2011	6		1.93	1.55–2.41	*	Genetic
Tian et al., 2011	4	1.26		0.95–1.57	*	Treatment
Trzesniak et al., 2011	11		1.98	1.33–2.94	*	Brain imaging
Veehof et al., 2011	8	0.37		0.20–0.53	*	Treatment
Vergouwen et al., 2011	24		0.83	0.74–0.93	*	Treatment
Vieta et al., 2011	10		0.68*^a^*	0.60–0.77	*	Treatment
Wisdom et al., 2011	53	−0.14		−0.21 to −0.07	*	Genetic
Witteman et al., 2011	26	−1.41		−1.76 to −1.05	*	Psychology
Woon and Hedges, 2011	24	−0.60		−0.83 to −0.37	*	Brain imaging
Xuan et al., 2011	20		1.00	0.86–1.16	—	Genetic
Yang et al., 2011a, cohort	14		1.38*^a^*	1.18–1.61	*	Miscellaneous
Yang et al., 2011a, case–control	7		2.48	1.93–3.19	*	Miscellaneous
Yang et al., 2011b	3	0.67		0.43–0.92	*	Treatment
Yuan et al., 2011	14		4.98	3.97–6.23	*	Genetic
Zafar et al., 2011	8		1.07*^a^*	0.91–1.27	—	Treatment
Zhang et al., 2011	12		1.27	1.01–1.59	*	Genetic
Zhu et al., 2011	8	0.84		0.18–1.49	*	Brain imaging

Classification performed by two independent raters.

*^a^*Relative risk.

*p < 0.05.

For experiments with a binary outcome, power was calculated by assuming that the expected incidence or response rate for the control group (i.e., the base rate) was equal to that reported in the corresponding meta-analysis and, similarly, used an assumed “treatment effect” (odds or risk ratio) equal to that given by each meta-analysis. The test statistic used for the calculation was the log odds-ratio divided by its SE. The latter was derived from a first-order approximation and estimated by the square root of the sum of the reciprocals of the expected values of the counts in the 2-by-2 summary table. The test statistic itself was then referenced to the standard normal distribution for the purposes of the power calculation. For studies reporting Cohen's *d*, the assumed treatment effect was again taken directly from the corresponding meta-analysis and all power calculations were based on the standard, noncentral *t* distribution. For comparability with the original study, we calculated the median power across all 730 individual studies which was equal to 23%, close to the 21% reported by Button et al. (2013).

**Figure 1.**
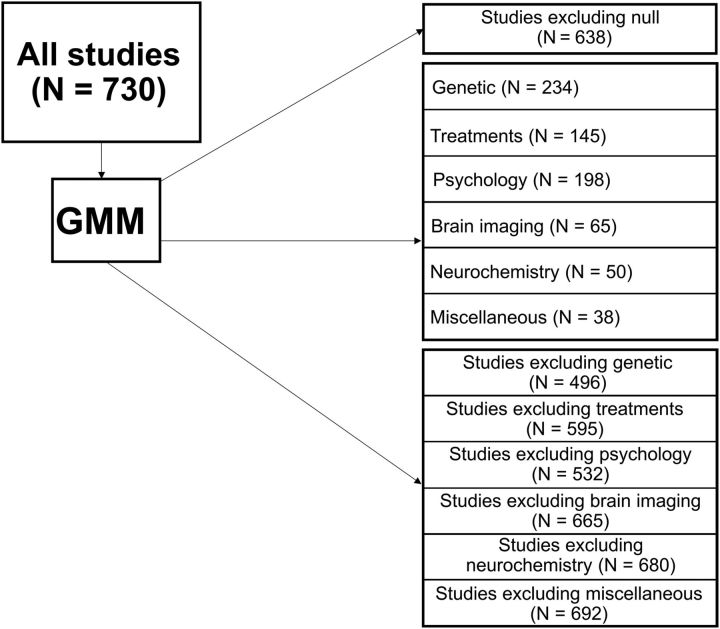
Classification of studies for analysis. Description of study methodology. GMM = Gaussian mixture model.

Figure 1 shows an overview of our analytical process. We additionally classified each study according to methodology: candidate gene association studies (*N* = 234), psychology (*N* = 198), neuroimaging (*N* = 65), treatment trials (*N* = 145), neurochemistry (*N* = 50), and a miscellaneous category (*N* = 38 studies from *N* = 2 meta-analyses). Two independent raters categorized the 49 meta-analyses into these six subfields, with 47/49 classified consistently; the remaining two were resolved after discussion. Before continuing our analysis in more depth, we present the reader with results that are directly comparable to the analysis of Button et al. (2013) (with the addition of the subfields; Table 2). These results are intended for comparison with our more nuanced characterization of the distributions using GMMs presented below; given the results of those GMMs (which suggest the these distributions are multimodal and therefore not well characterized by a single measure of central tendency), they should not be used to draw strong inferences.

**Table 2.** Median, maximum, and minimum power subdivided by study type

Group	Median power (%)	Minimum power (%)	Maximum power (%)	2.5^th^ and 97.5^th^ percentile (based on raw data)	95% HDI (based on GMMs)	Total N
All studies	23	0.05	1	0.05–1.00	0.00–0.72, 0.80–1.00	730
All studies excluding null	30	0.05	1	0.05–1.00	0.01–0.73, 0.79–1.00	638
Genetic	11	0.05	1	0.05–0.94	0.00–0.44, 0.63–0.93	234
Treatment	20	0.05	1	0.05–1.00	0.00–0.65, 0.91–1.00	145
Psychology	50	0.07	1	0.07–1.00	0.02–0.24, 0.28–1.00	198
Imaging	32	0.11	1	0.11–1.00	0.03–0.54, 0.71–1.00	65
Neurochemistry	47	0.07	1	0.07–1.00	0.02–0.79, 0.92–1.00	50
Miscellaneous	57	0.11	1	0.11–1.00	0.09–1.00	38

We also provide the 2.5^th^ and 97.5^th^ percentile of the frequency distribution of power estimates of individual studies for the raw data and 95% highest-density intervals (95% HDI) for the GMMs. We used HDIs to summarize the intervals of the most probable values from the distribution. HDIs differ from CIs in that they represent the most probable values of the distribution rather than symmetric credible intervals in a central tendency. As a result, HDIs are more suitable for summarizing skewed and multimodal distributions than CIs. HDIs were computed using the HDRCDE R toolbox, which finds the shortest intervals such that these intervals encompass the 95% most probable values of the distribution. Multiple intervals may be identified if a region between modes of the distribution is unrepresentative of the distribution (i.e. below the 5% threshold) (Wand et al., 1991; Hyndman, 1996; Samworth and Wand, 2010), which occurs for multimodal data.

### One or many populations?

The common measures of central tendency (mean, median, and mode) may not always characterize populations accurately because distributions can be complex and made up of multiple “hidden” subpopulations. Consider the distribution of height in the United States: the mean is 168.8 cm (Fryar et al., 2012). This statistic is rarely reported because the distribution comprises two distinct populations: male (175.9 cm, 5^th^–95^th^ percentile 163.2–188.2 cm) and female (162.1 cm, 5^th^–95^th^ percentile 150.7–173.7 cm). The mean of the male population is greater than the 95^th^ percentile of the female population. Therefore, a single measure of central tendency fails to describe this distribution adequately.

In an analogous fashion, the original study by Button et al. (2013) reported a median of 21% power, which could be interpreted as implying a degree of statistical homogeneity across neuroscience. The use of the median as a summary statistic while having the straightforward interpretation of “half above and half below” also implies that the power statistics are drawn from a distribution with a single central tendency. As we show below, this assumption is contradicted by our analyses, which makes the median statistic difficult to interpret. It should be noted that Button et al. (2013) themselves described their results as demonstrating a “clear bimodal distribution.” Therefore we explored the possibility that the power data originated from a combination of multiple distributions using GMM.

**Figure 2.**
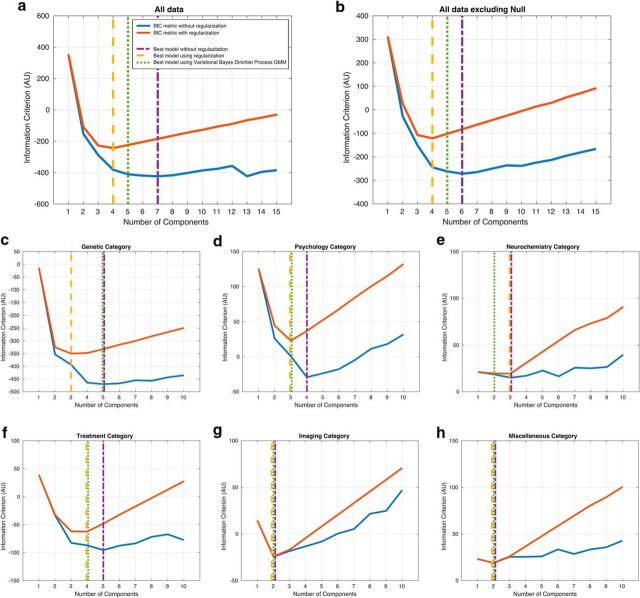
Model comparison and model selection analysis for GMMs, regularized GMMs, and DPGMMs. The blue and red lines display BIC scores (natural log scale) for nonregularized GMMs and regularized GMMs, respectively, for different levels of model complexity (number of mixture components). The lowest BIC score indicates the model that provides the best compromise between model fit (likelihood) and model complexity for the given dataset. Winning models for GMMs (purple dotted-dash vertical line), regularized GMMs (yellow dashed vertical line), and DPGMMs (green dotted vertical line) are clearly present for each dataset, enabling direct comparison of the output for each methodology. The regularized GMM approach provided the most parsimonious interpretation of the data on the two main datasets: all studies (***a***), excluding null studies (***b***) as well as five out of six subfield datasets (***c***–***h***).

GMM (similar to latent class analysis and factor models; Lubke and Muthén, 2005) can be used to represent complex density functions in which the central limit theorem does not apply, such as in the case of bimodal or multimodal distributions. We fit GMMs with varying numbers of *k* unknown components to the data and performed model selection using Bayesian information criteria (BIC) scores to compare models with different fit and complexity (i.e., the higher the number of *k* unknown components, the more complex the model). This allowed us to take a data-driven approach, as opposed to direct mixture models using a set number of components: therefore, we were agnostic as to the number of components that emerged from the model. The GMM with the lowest BIC identifies the most parsimonious model, trading model fit against model complexity. A difference in BIC between models of 10 or above on a natural logarithm scale is indicative of strong evidence in support of the model with the lower score (Kass and Raftery, 1995). To ensure that we used the most suitable GMM for this dataset, we ran different GMM models: standard GMMs, regularized GMMs, and Dirichlet process GMMs (DPGMMs; see below for full methods and Fig. 2 for model comparison and selection). The results were similar using each of these techniques (Fig. 2).

#### 

##### Finite Gaussian mixture model.

For a finite GMM, the corresponding likelihood function is given by the following (Corduneanu and Bishop, 2001):

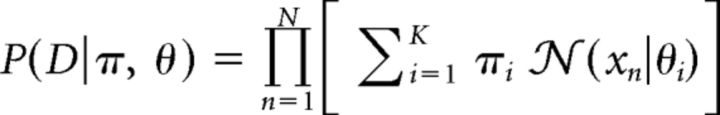
 where π_i_ denotes the mixing coefficient (proportions of the *i*th component), 𝒩(*x_n_*|θ*_i_*) denotes the conditional probability of the observation *x_n_* given by a Gaussian distribution with parameters θ*_i_*, and *D* denotes the whole dataset of observations, *x_n_*. Generally speaking, this means that we believe that there is an underlying generative structure to the observed data and that a mixture of Gaussian components would be a reasonable description/approximation of the true generative process of these data. That is, we assume that the data *D* have been generated from a mixture of Gaussian distributions with varying means, variances, and weights (model parameters), which we want to uncover. To do so, we perform model inversion and find the point estimates of the model parameters that maximize the likelihood (see the equation above) of the observed data (maximum likelihood estimation).

Model inversion is performed using the iterative expectation–maximization algorithm, which finds a local maximum of the likelihood function given initial starting parameters. We performed 50 restarts with kmeans++ initialization (Arthur and Vassilvitskii, 2007). Multiple restarts were performed to find the global maximum of the likelihood (i.e., the best GMM for the data: the parameters that maximize the chance of observing the data), as opposed to a local maximum. This allowed us to ensure that convergence was achieved for all GMMs on all datasets.

Traditionally, finite mixture modeling approaches require the number of components to be specified in advance of analyzing the data. That is, for each finite Gaussian mixture model fitted to the data, one is required to input the number of components *K* present in the mixture (model inversion only estimates the parameters for each component). Finding the number of components present in the data is a model selection problem and requires fitting multiple GMMs with varying numbers of components to the data, comparing the model evidence for each fit, and selecting the most parsimonious model for the data in question (Bishop, 2006; Gershman and Blei, 2012; Murphy, 2012).

It is worth noting, however, that GMMs can be subject to instabilities such as singularities of the likelihood function. Specifically, it is possible for one component to “collapse” all of its variance onto a single data point, leading to an infinite likelihood (Bishop, 2006; Murphy, 2012), and to incorrect parameter estimation for the model. Multiple techniques have been developed to address this problem. The simplest and most commonly used technique is to introduce a regularization parameter. Another is to adopt a fully Bayesian approach and apply soft constraints on the possible range of likely parameter values, therefore preventing problematic and unrealistic parameter values. Both methodologies were used in this study and we report on the resulting analysis for both implementations in the model selection section (below).

##### Finite Gaussian mixture model with regularization.

In typical finite mixture models, a regularization parameter can be added to avoid likelihood singularities. To do so, a very small value is added to the diagonal of the covariance matrix, enforcing positive-definite covariance and preventing infinitely small precision parameters for individual components. This model specification enables one to address the issue of “collapsing” components, but also enforces simpler explanations of the data, favoring models with fewer components. The larger the regularization parameter, the simpler the models will be, because single components will tend to encompass a larger subspace of the data partition. In this study, we introduced a regularization parameter of 0.001, which represents a reasonable trade-off between preventing overfitting components to noise in the dataset while capturing the most salient features from the data (the separate peaks), thus providing a better generative model of the data than using nonregularized GMMs. We used this approach for our primary inferences.

##### Dirichlet process Gaussian mixture model.

DPGMMs are a class of Bayesian nonparametric methods that avoid the issue of model selection when identifying the optimal number of components in a mixture model (Gershman and Blei, 2012; Murphy, 2012). With DPGMMs, we expand the original GMM model to incorporate a prior over the mixing distribution and a prior over the component parameters (mean and variance of components). Common choices for DPGMM priors are conjugate priors such as the normal-inverse-Wishart distribution over the mean and covariance matrix of components and a nonparametric prior over mixing proportions based on the DP.

The DP, often referred to as the Chinese restaurant process or the stick-breaking process, is a distribution over infinite partitions of integers (Gershman and Blei, 2012; Murphy, 2012). As a result, the DPGMM theoretically allows for an infinite number of components because it lets the number of components grow as the amount of data increases. The DP assigns each observation to a cluster with a probability that is proportional to the number of observations already assigned to that cluster. That is, the process will tend to cluster data points together, depending on the population of the existing cluster and a concentration parameter α. The smaller the α parameter, the more likely it is that an observation will be assigned to an existing cluster with probability proportional to the number of elements already assigned to this cluster. This phenomenon is often referred to as the “rich get richer.” This hyperparameter α indirectly controls how many clusters one expects to see from the data (another approach is to treat α as unknown, using a gamma hyperprior over α, and letting the Bayesian machinery infer the value; Blei and Jordan, 2006).

Implementation and analysis for the nonregularized finite GMMs, regularized finite GMMs, and DPGMMs was performed using MATLAB R2015b (The MathWorks) using the Statistics and Machine Learning toolbox, the Lightspeed toolbox, and the vdpgm toolbox (Kurihara et al., 2007).

### Model selection

To identify the winning model we used the BIC, which allows one to compute an approximation to the Bayes factor (relative evidence) for a model. The BIC typically has two terms, the likelihood (how well the model fits the data) and a complexity term that penalizes more complex models with more free parameters (e.g., the number of components). The model with the lowest BIC metric is usually preferred because it provides the most parsimonious and generalizable model of the data.

For each one of the following datasets, model fits were performed using nonregularized and regularized finite mixtures with up to 15 components (up to 10 components for the subfield categories; Fig. 2): the original dataset; the original dataset excluding null studies; each methodological subfield within the original dataset (genetics, psychology, neurochemistry, treatment, imaging, and miscellaneous studies); and the original dataset excluding each methodological subfield. Model selection was then performed using the BIC to select the most parsimonious model for each dataset. Figure 2 presents (for each dataset) the corresponding BIC metric for increasing levels of model complexity. Plain blue lines denote the BIC metric using nonregularized GMMs and plain red lines denote the BIC using regularized GMMs. The BIC metric curve for nonregularized GMMs (blue line) exhibits wide jumps (Fig. 2), whereas the function should remain relatively smooth, as seen with regularized GMMs (red line). This suggests that nonregularized GMMs results were prone to overfitting and were inadequate for some of our datasets.

Finally, we compared different modeling methodologies to select and report the most robust findings in terms of the estimation of the number of components. We compared nonregularized GMMs, regularized GMMs, and DPGMMs on the same datasets (Fig. 2) and found that regularized GMMs generally provided the most conservative estimation of the number of components. We therefore opted to report these results as the main findings.

## Results

**Figure 3.**
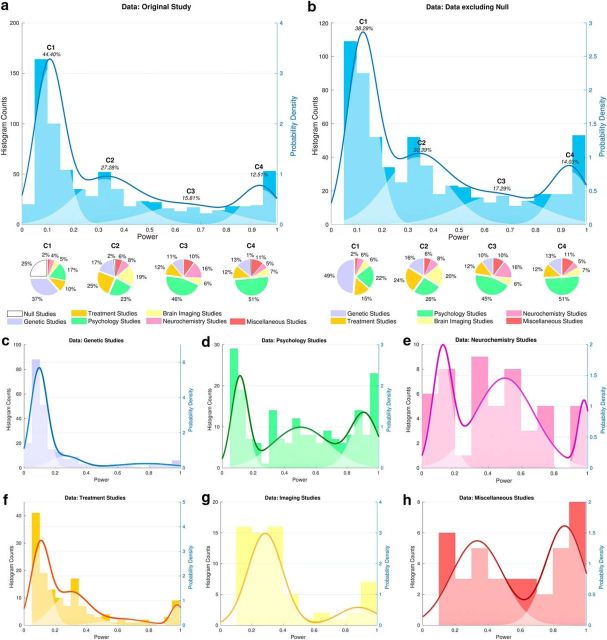
Power of studies. Shown are histograms depicting the distribution of study powers across all 730 studies (***a***) and across studies excluding null meta-analyses (***b***). However, we note that excluding power statistics from studies included in null meta-analyses may provide an overestimation of power because, in many instances, there remains uncertainty as to whether a true effect exists. Pale overlay shows the results of the regularized GMM, identifying four components (C1, C2, C3, and C4) and their relative weights within the dataset. Below the histogram, pie charts depict methodological subfields and null meta-analyses contributing to each component. The null studies (white pie-chart sections) comprise 52 genetic studies and 40 treatment studies. The dark blue line shows the sum of the components (overall GMM prediction). ***c***–***h***, Histograms depicting the distribution of study powers across all meta-analyses separated by subfield: candidate gene association studies (***c***), psychology studies (***d***), neurochemistry studies (***e***), treatment studies (***f***), imaging studies (***g***), and miscellaneous studies (***h***). Pale overlays show the results of the regularized GMM for each subfield; the dark lines show the sum of the components (overall GMM prediction).

We analyzed the original sample of 730 powers (see histogram in Fig. 3*a*). If the median were the most appropriate metric to describe the distribution of powers across studies, then we would expect the GMM to produce a solution containing only a single component. Instead, the most parsimonious GMM solution included four components, with strong evidence in favor of this model versus either of the next best models (i.e., GMMs with three or five components; Fig. 2). Importantly, this model revealed that the overall distribution of power appears to be composed of subgroups of lower- and higher-powered studies (Fig. 3*a*, overlay). We next explored possible sources of this variability, considering the influence of both null effects and specific subfields of neuroscience.

### When is an effect not an effect?

The first important source of variability that we considered relates to the concept of power itself. The calculation of power depends not just on the precision of the experiment (heavily influenced by the sample size), but also on the true population effect size. Logically, power analysis requires that an effect (the difference between population distributions) actually exists. Conducting a power analysis when no effect exists violates this predicate and will therefore yield an uninterpretable result. Indeed, when no effect exists, the power statistic becomes independent of the sample size and is simply equal to the type I error rate, which by definition is the probability of declaring a significant result under the null hypothesis.

To illustrate this point, consider the meta-analysis titled “No association between APOE ε 4 allele and multiple sclerosis susceptibility” (Xuan et al., 2011), which included a total of 5472 cases and 4727 controls. The median effect size (odds ratio) reported was precisely 1.00, with a 95% confidence interval (CI) from 0.861 to 1.156. Button et al. (2013) calculated the median power to be 5%, which is equal to the type I error rate. However, as is evident from the study's title, this meta-analysis was clearly interpreted by its authors as indicating a null effect, which is consistent with the observed result. Indeed, in this case, the power is 5% for both the largest (*N* > 3000) and the smallest (*N* < 150) study in the meta-analysis. In such cases, the estimate of 5% power is not easily interpretable.

Conversely, it is problematic to assume that a nonsignificant meta-analytic finding can be taken as evidence that there is no true effect; in the frequentist statistical framework, failure to reject the null hypothesis cannot be interpreted as unambiguous evidence that no effect exists (due to the potential for false-negative results). For example, the study by Chung and Chua (2011) entitled “Effects on prolongation of Bazett's corrected QT interval of seven second-generation antipsychotics in the treatment of schizophrenia: a meta-analysis” reported a median effect size (odds ratio) of 0.67, with a 95% CI from 0.43 to 1.04. Although this result was nonsignificant, the point estimate of the effect size is greater than those from several meta-analyses that did achieve statistical significance and, in our view, it would be premature to conclude that this effect does not exist.

These examples illustrate the difficulty in deciding whether conducting a power analysis is appropriate. Even tiny effect sizes could hypothetically still exist: in any biological system, the probability that an effect is precisely null is itself zero; therefore, all effects “exist” by this definition (with certain exceptions, e.g., in the context of randomization), even if to detect them we might need to test more individuals than are currently alive. However, the notion of “falsely rejecting the null hypothesis” then loses its meaning (Jacob Cohen, 1994). One approach would be to assume that an effect does not exist until the observed evidence suggests that the null hypothesis can be rejected, consistent with the logical basis of classical statistical inference. This would avoid any potential bias toward very-low-power estimates due to nonexistent effects. Conversely, this approach raises the potential problem of excluding effects that are genuinely very small, which may cause a bias in the other direction. Within the constraints of the null hypothesis significance testing framework, it is impossible to be confident that an effect does not exist at all. Therefore, we cannot simply assume that an effect does not exist after failing to reject the null hypothesis because a small effect could go undetected.

Motivated by this logic (specifically, that excluding power statistics from studies included in null meta-analyses may provide an overestimation of power because, in many instances, there remains uncertainty as to whether a true effect exists), we initially included studies from “null meta-analyses” (i.e., those in which the estimated effect size from the meta-analysis was not significantly different from the null at the conventional α = 0.05) in our GMMs (Fig. 3*a*). However, we note that excluding power statistics from studies included in null meta-analyses may provide an overestimation of power because, in many instances, there remains uncertainty as to whether a true effect exists. Nonetheless, with the above caveats in mind, we also wished to assess the degree to which null meta-analyses may have affected the results. Null results occurred in seven of the 49 meta-analyses (92 of the 730 individual studies), contributing a substantial proportion of the extremely low-powered studies (<10% power; Fig. 3*a*, white pie chart segment of C1). When we restricted our analysis only to studies within meta-analyses that reported statistically significant results (“non-null” meta-analyses), the median study power (unsurprisingly) increased, but only slightly, to 30%, and the nature of the resulting GMM distribution did not change substantially (Fig. 3*b*). In other words, excluding null meta-analyses does not provide a radically different picture. Therefore, we also examined another potential contributor to power variability in neuroscience: the influence of specific subfields of neuroscience.

### Power in neuroscience subfields

As described above, we categorized each meta-analysis into one of six methodological subfields. Interestingly, statistical power varied significantly according to subfield (permutation test of equivalence: *p* < 0.001), with genetic association studies lower (11% median power) than any other subfield examined (all *p* < 0.001, Mann–Whitney *U* tests). Such variability across neuroscience subfields is consistent with the original report by Button et al. (2013), which reported the median power of animal studies (18% and 31% for two meta-analyses) and case-control structural brain imaging studies (8% across 41 meta-analyses). However, even within specific subfields, the distribution of power is multimodal (Fig. 3*c–h*). This could represent variability in statistical practices across studies, but another possible explanation is that the size of the effect being studied varies substantially between meta-analyses, even within the same subfield. This alternative explanation may, at least in part, account for the variability between (and within) subfields of neuroscience.

The large number of extremely low-powered candidate gene association studies warrants additional comment. These were included in the original analysis because the Web of Science classifies such studies as “neuroscience” if the phenotypes in question are neurological or psychiatric disorders. However, modern genome-wide association studies have revealed that the overwhelming majority of candidate gene association studies have been underpowered because the reliable associations that have been identified are extremely weak (Flint and Munafò, 2013); therefore, very low power is expected within this subgroup, which our analysis confirms (Fig. 3*c*). This subgroup of studies can offer important lessons to the rest of neuroscience: without large genetic consortia, the field of neuropsychiatric genetics might still be laboring under the misapprehension that individual common variants make substantial contributions to the risk for developing disorders. Providing that sampling and measurement are standardized, pooling data across multiple sites has the potential to improve dramatically, not only statistical power, but also the precision on estimates of effect size.

**Figure 4.**
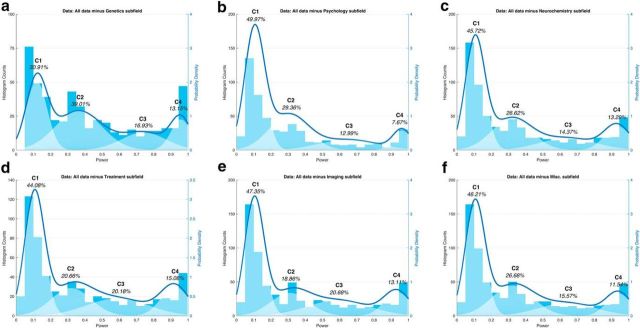
GMMs excluding each subfield. GMMs for the whole population of studies excluding genetic studies (***a***), psychology studies (***b***), neurochemistry studies (***c***), treatment studies (***d***), imaging studies (***e***), and the remaining miscellaneous studies (***f***). Compare with the distribution including all studies (Fig. 3*a*).

Because numerous studies report that candidate gene association studies are severely underpowered (Klerk et al., 2002; Colhoun et al., 2003; Duncan and Keller, 2011), and given that candidate gene association studies comprised more than one-third of our total sample of studies, we suspected that they might contribute heavily to the lowest-power peak in our distribution. We confirmed this: in the absence of genetic studies, many studies remained underpowered, but the distribution contained proportionally fewer studies in the lowest-power peak (∼10% power; Fig. 4*a*). Although low power is clearly not limited to candidate gene association studies, they have a greater influence on the overall power distribution than any other subfield, skewing the distribution toward the lowest-power peak (Fig. 4*b–f*).

### Simulating power in hypothetical fields

**Figure 5.**
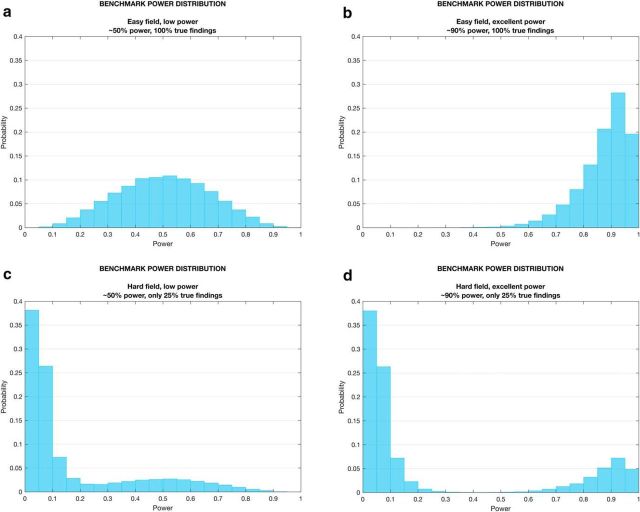
Simulated power distributions for four hypothetical fields: “easy field” with low power (∼0.5) and all effects exist (***a***); “easy field' with high power (∼0.9) and all effects exist (***b***); “hard field” with low power (∼0.5) (for those effects that exist), but where effects exist in only 25% of cases (***c***); and “hard field” with high power (∼0.9) (for those effects that exist), but where effects exist in only exist in 25% of cases (***d***). Power distributions were simulated by generating 50,000 power estimates for a one sample t-test with a fixed sample size (*N* = 45) while varying effect size. For each panel, the effect size was sampled from a truncated (effect size >0) Gaussian distribution with mean 0.3 (***a***, ***c***) or 0.49 (***b***, ***d***) to represent low or high power, respectively. For the “hard” fields (***c***, ***d***), 75% of the effect size sample was generated from a half-Gaussian distribution with mean = 0. SD was set to 0.07 for all effect size distributions. Similar results can be obtained by fixing the effect size and varying the sample size.

One clear conclusion of our analyses is that the interplay between the proportion of true effects and the power to detect those effects is crucial in determining the power distribution of a field. We simulated four power graphs for hypothetical fields to illustrate this point: one with low power (∼50%) in which all effects exist (Fig. 5*a*); one with high power (∼90%) in which all effects exist (Fig. 5*b*); one with low power (∼50%), in which only a minority (25%) of effects exist (Fig. 5*c*); and one with high power (∼90%) in which only a minority (25%) of effects exist (Fig. 5*d*). We found that the “low-power” field did not resemble the distribution of power in neuroscience that we observed (Fig. 3*a*). Instead, our findings were closest to a mixture of two distributions: Figure 5*c* with low (∼50%) power in which only 25% of findings are true effects and Figure 5*d* with high (∼90%) power in which only 25% of findings are true effects. This would be consistent with the notion that the absence of true effects may contribute to the distribution of statistical power in neuroscience.

## Discussion

### Implications for neuroscience

We argue that a very influential analysis (cited >1500 times at the time of writing) does not adequately describe the full variety of statistical power in neuroscience. Our analyses show that the dataset is insufficiently characterized by a single distribution. Instead, power varies considerably, including between subfields of neuroscience, and is particularly low for candidate gene association studies. Conducting power analyses for null effects may also contribute to low estimates in some cases, though determining when this has occurred is challenging. Importantly, however, power is far from adequate in every subfield.

Our analyses do not negate the importance of the original work in highlighting poor statistical practice in the field, but they do reveal a more nuanced picture. In such a diverse field as neuroscience, it is not surprising that statistical practices differ. Whereas Button et al. (2013) were careful to point out that they identified a range of powers in neuroscience, their reporting of a median result could be interpreted as implying that the results were drawn from a single distribution, which our analyses suggest is not the case. We confirm that low power is clearly present in many studies and agree that focusing on power is a critical step in improving the replicability and reliability of findings in neuroscience. However, we also argue that low statistical power in neuroscience is neither consistent nor universal.

Ethical issues accompany both underpowered and overpowered studies. Animal deaths, drugs taken to human trials, and government funding are all wasted if power is too low. However, blindly increasing sample size across the board simply to satisfy concerns about field-wide power failures is also not the best use of resources. Instead, each study design needs to be considered on its own merits. In this vein, one response to the original article pointed out that any measure of a study's projected value suffers from diminishing marginal returns: every additional animal or human participant adds less statistical value than the previous one (Bacchetti et al., 2005, 2008, 2013).

Studies with extremely large sample sizes can also fall prey to statistically significant findings for trivial effects that are unlikely to be either theoretically or clinical important (Lenth, 2001; Ioannidis, 2005; Friston, 2012; Quinlan, 2013). In other words, the assessment of power is determined by what we consider to be an interesting (i.e., nontrivial) effect size (Cohen, 1988). This dependency means that power considerations are meaningless in the absence of assumptions about how large effect sizes need to be to be considered theoretically or clinically important and this may vary dramatically across different fields. This is particularly relevant in fields in which multiple comparisons are performed routinely, such as genetics and neuroimaging (Friston, 2012). Conversely, smaller studies can only detect large effect sizes and may suffer from imprecise estimates of effect size and interpretive difficulties. Crucially, there is no single study design that will optimize power for every genetic locus or brain area. In fact, power estimates for individual studies are themselves extremely noisy and may say little about the actual power in any given study. A move away from presenting only *p*-values and toward reporting point estimates and CIs (as long advocated by statisticians) and toward sharing data to improve such estimates would allow researchers to make better informed decisions about whether an effect is likely to be clinically or theoretically useful.

### Estimations of effect size

An important factor contributing to the estimation of power (at least using the approach followed here) is whether the effect size was estimated accurately *a priori*. If researchers initially overestimated the effect size, then even the sample size specified by a power calculation would be insufficient to detect a real, but smaller effect. Interestingly, our analysis also shows the existence of very-high-powered studies within neuroscience, in which far more subjects have been included than would technically be warranted by a power analysis. In this case, an *a priori* underestimate of effect size could yield a very-high-powered study if an effect proves to be larger than initially expected (which has occasionally been reported; Open Science Collaboration, 2015). Another important consideration is that an overestimation of effect size might occur due to publication bias, which will skew effect size estimates from meta-analyses upwards, resulting in an optimistic power estimate. This is an important caveat to the results that we report here: a bias toward publishing significant results means that the power estimates that we report will represent upper bounds on the true power statistics. Unfortunately, we could not adequately address this potential confound directly because tests of publication bias themselves have very low power, particularly if the number of studies in a meta-analysis is low. However, publication bias has long been reported in psychology (Francis, 2012) and neuroscience (Sena et al., 2010), so it is reasonable to assume that it has inflated estimates of statistical power in these analyses.

### Conclusion

We have demonstrated the great diversity of statistical power in neuroscience. Do our findings lessen concerns about statistical power in neuroscience? Unfortunately not. In fact, the finding that the distribution of power is highly heterogeneous demonstrates an undesirable inconsistency both within and between methodological subfields. However, within this variability are several appropriately powered and even very-high-powered studies. Therefore, we should not tar all studies with the same brush, but instead should encourage investigators to engage in the best research practices, including preregistration of study protocols (ensuring that the study will have sufficient power), routine publication of null results, and avoiding practices such as *p*-hacking that lead to biases in the published literature.

## Notes

Supplemental material for this article is available at http://osf.io/duyxb. Available are open data, including analysis scripts and meta-analysis data. This material has not been peer reviewed.
